# Diabetes screening with hemoglobin A1c prior to a change in guideline recommendations: prevalence and patient characteristics

**DOI:** 10.1186/1471-2296-12-91

**Published:** 2011-08-24

**Authors:** Michelle Greiver, Babak Aliarzadeh, Rahim Moineddin, Christopher Meaney, Noah Ivers

**Affiliations:** 1 Department of Family and Community Medicine, University of Toronto, 500 University Avenue, Toronto, Ontario, M5G 1V7, Canada; 2 North Toronto Research Network, 240 Duncan Mill Road, Suite 705 Toronto, Ontario, M3B 3S6, Canada; 3 Department of Family and Community Medicine, University of Toronto, 500 University Avenue, Toronto, Ontario, M5G 1V7 Canada; 4 Institute for Clinical Evaluative Sciences, 2075 Bayview Avenue, Toronto, Ontario, M4N 3M5, Canada; 5 Dalla Lana School of Public Health, University of Toronto, 155 College Street, Toronto, Ontario, M5T 3M7, Canada; 6 Research Program, Department of Family and Community Medicine, University of Toronto, 500 University Avenue, Toronto, Ontario, M5G 1V7, Canada; 7 Women's College Hospital, 76 Grenville Street, Toronto Ontario, M5S 1B2, Canada; 8 Clinical Epidemiology, Department of Health Policy, Management and Evaluation, University of Toronto, 155 College Street, Suite 425, Toronto, Ontario, M5T 3M6, Canada

## Abstract

**Background:**

In January 2010, the American Diabetes Association recommended the use of hemoglobin A1c (Hgb A1c) to screen and diagnose diabetes. This study explored the prevalence and clinical context of Hgb A1c tests done for non-diabetic primary care patients for the three years prior to the release of the new guidelines. We sought to determine the provision of tests in non-diabetic patients age 19 or over, patients age 45 and over (eligible for routine diabetes screening), the annual change in the rate of this screening test, and the patient characteristics associated with the provision of Hgb A1c screening.

**Methods:**

We conducted a retrospective study using data routinely collected in Electronic Medical Records. The participants were thirteen community-based family physicians in Toronto, Ontario. We calculated the proportion of non diabetic patients who had at least one Hbg A1c done in three years. We used logistic generalized estimating equation with year treated as a continuous variable to test for a non-zero slope in yearly Hbg A1c provision. We modelled screening using multivariable logistic regression.

**Results:**

There were 11,792 non-diabetic adults. Of these, 1,678 (14.2%; 95%CI 13.6%-14.9%) had at least one Hgb A1c test done; this was higher for patients 45 years of age or older (20.2%; 95% CI 19.3% - 21.2%). The proportion of non-diabetic patients with an A1c test increased from 5.2% in 2007 to 8.8% in 2009 (p < 0.0001 for presence of slope). Factors associated with significantly greater adjusted odds ratios of having the test done included increasing diastolic blood pressure, increasing fasting glucose, increasing body mass index, increasing age, as well as male gender and presence of hypertension, but not smoking status or LDL cholesterol. Patients living in the highest income quintile neighbourhoods had significantly lower odds ratios of having this test done than those in the lowest quintile (p < 0.001).

**Conclusions:**

A large and increasing proportion of the non-diabetic patients we studied have had an Hgb A1c for screening prior to guidelines recommending the test for this purpose. Several risk factors for cardiovascular disease or diabetes were associated with the provision of the Hgb A1c. Early uptake of the test may represent appropriate utilization.

## Background

Diabetes is an increasingly prevalent condition in Canada, with serious effects on morbidity and mortality [1]. This condition can be present for up to seven years prior to diagnosis [2], leading to recommendations for periodic screening of asymptomatic individuals [1,3,4].

Guideline recommendations regarding screening and diagnosis of diabetes are currently evolving. In 2009 the use of hemoglobin A1c (Hgb A1c), which represents an index of the average plasma glucose level over several weeks, was recommended by an international committee for the diagnosis of diabetes [5]. In 2010, the American Diabetes Association (ADA) agreed [3,4,6]. The ADA suggested that Hgb A1c be considered as an acceptable test to diagnose diabetes, with a confirmed value of 6.5% or greater being diagnostic [3].

The Canadian Diabetes Association's 2008 guidelines do not recommend Hgb A1c for screening or diagnosis. Fasting Blood Glucose (FBG) is recommended for screening [1], and diagnosis depends on repeated elevated glucose tests or an abnormal Oral Glucose Tolerance Test (OGTT) [1]. However, the OGTT is not often used in Ontario [7]; it is inconvenient for patients and more variable than fasting blood glucose or Hgb A1c [8]. Hgb A1c may be a better test for screening than FBG because it is more replicable [9], and offers superior prognostic value for neuropathy, retinopathy [9], and cardiovascular disease [10]. Hgb A1c is equal to FBG as a predictor of diabetes [10], but is more convenient than FBG because it does not require fasting. However, Hgb A1c is more costly [11], and may be inaccurate in the setting of hemoglobinopathies [3]. As well, normal Hgb A1c levels may be higher in black persons [12].

Despite the lack of any guideline-based recommendations at the time, 6.0% of adults without diabetes had a Hgb A1c done (presumably for diabetes screening) in Ontario in 2005 [7]. While this does suggest that Hgb A1c is commonly used for screening in this context, the study was limited by the secondary use of administrative data, which lacks clinical information including laboratory values. The investigators could not identify several elements underlying the clinical context of these tests.

Therefore, we used electronic medical records (EMRs) of community based family physicians to investigate the prevalence and clinical context of Hgb A1c tests done for patients without diabetes prior to the release of the new guidelines. We examined the change in rates of Hgb A1c over the three years preceding the release of the 2010 ADA guidelines. In order to explore patient characteristics associated with early adoption of the Hgb A1c test for screening purposes, we extracted information on a variety of clinical risk factors associated with increased cardiovascular risk or diabetes from data available in the EMRs.

## Methods

### Participants

The Canadian Primary Care Sentinel Surveillance Network (CPCSSN) is Canada's first multi-disease EMR-based surveillance system [13]. The North Toronto Research Network (NorTReN) is one of nine networks currently participating in CPCSSN. NorTReN is a practice based primary care research network affiliated with the Department of Family and Community Medicine at the University of Toronto. Family physicians participating in NorTReN contribute their EMR data; an anonymized version of applicable data is sent to CPCSSN's central data repository from each site and is then de-identified and aggregated in a single national database. Posters informing patients about the study are present in the waiting rooms of participating practices, and patients can opt out. One patient has opted out of the NorTReN database, while 111 patients have opted out of the national CPCSSN database.

We obtained data from the Electronic Medical Records (EMRs) of community based family physicians in Toronto, Ontario. Thirteen family physicians participating in NorTReN who had used an EMR for three years or more and who currently contribute data to CPCSSN were included in this study. All physicians were community-based, were members of a Family Health Team [14], and had rostered practices in which patients formally enrolled with their physician, allowing the identification of practice panels [15,16]. A Family Health Team consists of a group of physicians paid largely by capitation (a set fee based on rostered patients' age and gender); physicians work in multidisciplinary practices, along with nurses, social workers and other allied health professionals. The physicians in this study used a single EMR (Nightingale on Demand^®^).

### Eligible patients and determination of diabetic status

The eligible population included all non-diabetic rostered patients age 19 and over who had seen their family physician at least once between January 1^st ^2007 and December 31^st ^2009 and were active as of December 31^st ^2009.

In order to capture tests that were ordered for screening purposes (and not for disease monitoring), we identified and excluded all known diabetic patients. To define diabetes, we followed a previously validated algorithm for administrative databases [17] and used two billing codes for diabetes in two years. This algorithm only had a sensitivity of 86% [17]. However, we are able to access data unavailable in administrative databases; our definition added the presence of diabetes in the problem list and we used free-text search terms that would likely maximize the number of true positives for the diagnosis of diabetes. If neither billing nor problem list data were present, we then searched for the presence of any current hypoglycemic medication (insulins, sulfonylureas, metformin, thiazolidinediones, incretins, or acarbose), or for an Hgb A1c of 7% or greater, or two fasting blood glucose tests of 7 mmol/l or greater [1]. We then manually excluded patients with polycystic ovarian syndrome (metformin can be used clinically for ovulation induction and management of metabolic syndrome in PCOS) [18-20], gestational diabetes, secondary diabetes, and hyperglycemia not otherwise specified based on coding for these conditions. The approach is similar to that of a validated algorithm using EMR data [21]. Further development of electronic data queries for case definitions had been undertaken as part of CPCSSN [13].

Laboratory screening increases with age [22]. Screening in younger age ranges is recommended only for patients deemed to be at higher risk of chronic diseases [1,4,11,23]. This may confound our comparisons of the screened and non-screened groups as screening in general may be more limited in younger groups. To limit this source of bias, we present descriptive data restricted to those age 45 and over by December 31^st ^2009. By that age, both Canadian and American guidelines recommend diabetic screening for patients at low risk [1,4]. Patients presenting for a periodic health exam may be more likely to have screening blood work [22,24,25], as are those with a chronic health condition. There may be systematic differences between those having screening bloodwork and those not having this done [22]. We used the presence of total cholesterol in the past three years as a marker of a blood test done for screening [22,26] or chronic cardiovascular disease management. We then also calculated the proportion of screening Hgb A1c tests done for patients age 45 or over who had at least one total cholesterol done, that is those presumed to have presented for screening bloodwork.

### Data elements

We extracted values for Hgb A1c, as well as for data associated with cardiovascular risk factors [26-29]. The following data elements, if present in the EMR in the past three years (January 1 2007 to December 31 2009), were extracted: age in years, gender, current smoking status (if available in the patient's health profile), Hgb A1c, LDL cholesterol, fasting glucose levels, weight, BMI, waist circumference and blood pressure. Age was calculated as 'test done date' minus July 1, birth-year for all patients with Hgb AIc test done. For those without an Hgb A1C test, age was calculated as 'study end date' of December 31^st ^2009 minus July 1, birth-year for all patients. For laboratory measurements, we chose the value occurring immediately prior to the screening Hgb A1c test. If someone was screened on multiple occasions, we considered time of most recent screen and determined values of predictors from this time point. For patients that were never screened, we considered values of the predictors from the most recent time point. The Postal Code Conversion File was used to assign neighbourhood income quintile to the patients' residential postal code.[15]

Data completeness in EMRs used in routine care may be problematic, as clinicians may neglect to enter data or data may be entered in fields that are not captured during searches; some laboratory tests may not be received electronically [30]. To give an indication of data completeness we have included the percentage of missing values by study variable and patient group in the results section.

### Analysis

We first calculated the proportion of non-diabetic patients who had at least one Hbg A1c done in the past three years. We then calculated the proportion of patients with at least one Hgb A1c test in each year of interest. We used logistic generalized estimating equation with year treated as a continuous variable to test for a non-zero slope in Hbg A1c provision.

We compared characteristics of patients age 45 and over who did and did not have at least one Hgb A1c in the three years of interest. We used descriptive statistics such as means, medians, ranges, standard deviations and percentages to summarize baseline data. Logistic regression was used to identify clinically relevant factors associated with Hgb A1c testing [31]. First, we considered odds ratios and p values for each of the univariate predictors of screening. We then modelled screening (multivariable/adjusted model) as a function of various biochemical, demographic and behavioural covariates. All tests were two tailed and p value of < 0.05 was considered statistically significant. We used SAS version 9.2 for the analysis and data manipulation.

This study was approved by the North York General Hospital's Research Ethics Board. All physicians provided signed, informed consent.

## Results

Thirteen family physicians contributed data. The mean year of graduation was 1981, with a range of 1967 to 2002. Five physicians were male, and twelve were Canadian graduates.

The number of active rostered patients age 19 and over was 13,112, and,11,792 patients (89.9%) were not diabetic. Of these non-diabetic patients, 1,678 (14.2%; 95% CI 13.6% - 14.9%) had at least one Hgb A1c test done in the three year period prior to the release of the new ADA guidelines. The percentage of non-diabetic adults who had a Hgb A1c done was 5.2% in 2007, 7.5% in 2008 and 8.8% in 2009; the presence of a slope was significant (p < 0.0001).

There were 6,786 non-diabetic patients age 45 or more; 1,372 (20.2%; 95% CI 19.3% - 21.2%) of those patients had a screening Hgb A1c done. Limiting to those patients who had screening blood work (based on presence of a total cholesterol value in the chart), resulted in 4,863 patients, of whom 1,298 (26.7%; 95% CI 25.5% - 27.9%) had a screening Hgb A1c.

The characteristics of patients age 45 and over are presented in Table 1. 81.8% of patients with a Hgb A1c present were age 45 and over, and 94.6% of those patients had at least one total cholesterol value in the chart. The median age for patients who had a screening Hgb A1c was 63 (IQR 55 - 73), while median age for those without the test was 57 (IQR 50 - 68). One-fifth (19.8%) of those screened had fasting blood glucose in the impaired range (6.0 - 6.9 mmol/L), while 5.4% of those not screened had a value in this range. Nearly half (47%) of those screened were hypertensive, compared to 30% of those not screened.

**Table 1:** Patient characteristics, limited to patients who were at least 45 years old

	Total non-diabetic patients*		Non-diabetic patients without a screening Hgb A1c from 2006 to 2009		Non-diabetic patients with at least one screening Hgb A1c from 2006 to 2009	
***Parameter***	***Value***	***% with no data***	***Value***	***% with no data***	***Value***	***% with no data***

**N (%)**	6786(100)		5414(80)		1372(20)	
**% Male**	34	0	33	0	44	0
**Mean age in years (median, IQR)**	61 (59, 51-70)	0	60 (57, 50-68)	0	64 (63, 55-73)	0
**% current smoker**	7	10	7	12	8	5
**Mean weight in Kg (SD)**	73.3(17.1)	11	72.1(16.7)	13	77.0(17.9)	6
Mean BMI** (SD)	26.9(5.2)	13	26.5(5.0)	14	28.0(5.6)	8
**%BMI > = 30**	21		19		29	
**Mean WC in cm (SD)**	90 (14)	67	89 (13)	66	96 (12)	70
**Mean sBP in mmHg (SD)**	123(15)	8	123(15)	9	125 (15)	5
**Mean dBP in mmHg (SD)**	76 (9)	8	75(9)	9	77(9)	5
**% Patients with HTN**	34		30		47	
**Mean LDL cholesterol in mmol/l (SD)**	3.3(0.9)	23	3.3(0.9)	28	3.2(1.0)	5
**Mean FBG in mmol/l (SD)**	5.3(0.6)	23	5.2 (0.5)	29	5.5(0.6)	4
**% with FBG 6 to 6.9**	8.7		5.4		19.8	

*Patients indicated as being active and rostered to a participating physician on December 31^st ^2009, with at least one visit between January 1^st ^2007 and December 31^st ^2009

**BMI: Body mass index (weight in kilograms divided by height in meters squared)

WC: waist circumference; sBP: sytolic blood pressure; dBP: diastolic blood pressure; HTN: hypertension; FBG: fasting blood glucose; SD: standard deviation; IQR: interquartile range

Missing values differed between the groups. For example, 5% of those screened with Hgb A1c did not have a LDL cholesterol value during the three years for the measures we obtained, while 28% of those not screened had no value. Missing vital signs (other than waist circumference) ranged from 5% to 8% in those screened, and from 9% to 14% in those not screened.

Table 2 depicts the bivariate odds ratios associated with each possible predictor of being screened for patients age 45 and over. Increasing BMI, increasing waist circumference, increasing fasting glucose, increasing blood pressure, increasing age, male gender and presence of hypertension were associated with significantly greater bivariate odds ratios for being screened with Hgb A1c. Patients living in the highest income neighbourhood had significantly lower odds ratios compared to patients living in the lowest income neighbourhood for being screened. Smoking status was not associated with significantly different odds ratios for being screened with Hgb A1c.

**Table 2:** Bivariate results from logistic regression analysis of HgBA1C screening limited to patients who were at least 45 years old

	OR	95% CI (OR)	P-value	Sample Size (N)
**LDL cholesterol**	0.93	0.86 - 0.99	0.04	4,830
**BMI**	1.06	1.04 - 1.07	< 0.0001	5,192
**Waist Circumference**	1.05	1.04 - 1.06	< 0.0001	1,952
**Systolic blood pressure**	1.01	1.01 - 1.01	< 0.0001	5,619
**Diastolic blood pressure**	1.02	1.01 - 1.03	< 0.0001	5,614
**Fasting blood glucose**	2.66	2.36 - 3.00	< 0.0001	4,811
**Age**	1.03	1.02 - 1.03	< 0.0001	6,786
**Sex**				
• **Female**	---	---	---	---
• **Male**	1.77	1.57 - 1.99	< 0.0001	6,786
**Smoking Status**				
• **Current smoker**	---	---	---	---
• **Ex-Smoker**	1.22	0.95 - 1.57	0.12	6,088
• **Never Smoked**	0.94	0.75 - 1.19	0.62	
**Hypertension**				
• **No**	---	---	---	---
• **Yes**	2.15	1.91 - 2.43	< 0.0001	6,786
Neighborhood income quintile*				
• **1**	---	---	---	---
• **2**	0.81	0.64-1.02	0.07	6,617
• **3**	0.90	0.73-1.11	0.34	
• **4**	0.75	0.61-0.93	0.007	
• **5**	0.59	0.49-0.73	< 0.0001	

* The Postal Code Conversion File was used to assign neighbourhood income quintile to the patients' residential postal code; 1 indicates the highest income quintile, while 5 indicates the lowest income quintile

The multivariable/adjusted model is presented on table 3. It includes those factors found to be significant in the bivariate regression analysis, with the exception of LDL cholesterol and waist circumference. LDL cholesterol was excluded due to its marginal significance in the bivariate regression, while waist circumference was dropped from the model due to the high proportion of missing results. In the adjusted model, increasing diastolic blood pressure, increasing fasting glucose, increasing BMI, increasing age, as well as male gender and presence of hypertension were significantly associated with adjusted odds ratios of having a Hgb A1c done. Patients living in the highest income quintile neighbourhoods had significantly lower odds ratios of having this test done than those in the lowest income quintile.

**Table 3:** Adjusted Odds Ratios for the likelihood of being screened with Hgb A1c

	OR	95% CI(OR)	P-value
**Systolic blood pressure**	0.99	0.98 - 0.99	< 0.0001
**Diastolic blood pressure**	1.03	1.02 - 1.04	< 0.0001
**Fasting blood glucose**	2.22	1.91 - 2.57	< 0.0001
**BMI**	1.03	1.01 - 1.05	0.0002
**Age**	1.03	1.02 - 1.04	< 0.0001
**Sex**			
• **Female**	---	---	---
• **Male**	1.55	1.31 - 1.82	< 0.0001
**Hypertension**			
• **No**	---	---	---
• **Yes**	1.31	1.10 - 1.56	0.003
**Neighborhood income quintile**			
• **1**	---	---	---
• **2**	0.69	0.51 - 0.93	0.02
• **3**	0.83	0.63 - 1.10	0.20
• **4**	0.81	0.61 - 1.06	0.13
• **5**	0.63	0.48 - 0.82	0.001

## Discussion

The physicians in this study began using the Hgb A1c test for some non-diabetic patients well before the release of the guidelines; we found that a fifth of patients without diabetes age 45 or more had at least one Hgb A1c done in a three year period. The proportion of patients with an Hgb A1c test increased over time. It is encouraging to note that patients were more likely to be screened if they had clinical risk factors associated with diabetes, such as increasing age, higher fasting blood glucose, higher BMI, or presence of hypertension. In such patients, it is possible that Hgb A1c was added to fasting blood glucose to augment the detection of diabetes. The combination of the two tests can determine the presence of diabetes with greater sensitivity than a single test [32], but the effectiveness and cost-effectiveness of this approach is unknown [7].

Interestingly, female patients and patients at the highest socioeconomic levels had significantly lower odds ratios for being screened. A population-based study tracking Ontarians from 2000-2006 similarly found that non-diabetic patients were more likely to be screened with a blood glucose if they were hypertensive, had higher BMIs or were older; however, in that study, women were more likely to be screened than men [33]. The population-based analysis also found that the rate of Hgb A1c testing for non-diabetic patients in Ontario was rapidly increasing, and had reached 6.0% by 2005. In our study population, the rate was 8.8% in 2009. It is unclear if our findings represent unique features of our study population or changes over time in the utilization of Hgb A1c. Regardless, it seems likely that Hgb A1c utilization will accelerate if/when Canadian guidelines recommending Hgb A1c for screening are disseminated.

### Limitations

This study is limited to community-based family physicians participating in a Practice Based Research Network in Toronto and using a single EMR; results may not be applicable to other settings or other EMRs. Nevertheless, there is no a priori reason to believe that the use of Hgb A1c for screening would be systematically different in this group of physicians; therefore we believe the results from this study provide valuable insight into the gradual increase in Hgb A1c utilization.

Our criteria for determining the presence of diabetes are similar to those others have employed, but have not been formally validated. Validation of chronic disease criteria in the CPCSSN database is ongoing. Furthermore, we could not reliably include patients with an Hgb A1c of 7% or more into the screened cohort because data in the EMR were not complete enough to allow us to determine whether the diagnosis of diabetes existed prior to the test. Thus, there was uncertainty as to whether they were screened or monitored. Including patients with a Hgb A1c of 7% or more in the screening group would have inflated our results.

EMR data abstracted for this study were fairly complete, but there were differences in missing data between the populations screened and not screened with Hgb A1c. Nearly all (95%) of patients age 45 or over with a Hgb A1c screen had a LDL cholesterol present in the EMR. However, only 72% of those without Hgb A1c had at least one LDL cholesterol level present in the three years studied. We have no reasons to assume that rates of missing EMR data (test done, but data not present in an electronically auditable form) are different between the two groups, so it is possible that some patients did not have a Hgb A1c done simply because they did not present for any screening blood work.

This study examined factors extensively documented in the literature as being associated with cardiovascular risk factors [26-29] and clinically likely to influence a family physician's risk estimation. There are a large number of data elements in the EMR, and we wished to be conservative in order to exclude spurious associations that could be significant due to the large sample size. Therefore, we did not include LDL cholesterol in the model because it had only marginal statistical association and limited clinical plausibility. It is possible that consideration of other variables for the model may have altered the results, but in a sensitivity analysis, we included triglycerides and this did not change our findings.

## Conclusions

Hgb A1c screening was provided to patients by these community-based family physicians prior to the release of guidelines recommending the use of this test for screening purposes. The rates of screening increased over time. Patients screened had several clinical factors associated with greater risks of cardiovascular disease and diabetes, indicating that the utilization of Hgb A1c for screening in this group was frequently appropriate.

The incidence and prevalence of diagnosed diabetes may change due to the rising use of Hgb A1c screening and the introduction of Hgb A1c of 6.5% or greater as a new criterion for diagnosis [3]. In Ontario, it is estimated that 1.4% of the population is undiagnosed [33]. Should Canadian guidelines concur with American guidelines, a proportion of the undiagnosed practice populations could be immediately identified using data already present in the EMRs.

## Competing interests

The authors declare that they have no competing interests.

## Authors' contributions

MG, BA and NI conceived the study and its design. BA acquired the data from the EMRs. MG drafted the initial manuscript. RM and CM performed the analysis. All authors contributed to the interpretation of the data, revised the manuscript and read and approved the final manuscript.

## Pre-publication history

The pre-publication history for this paper can be accessed here:

http://www.biomedcentral.com/1471-2296/12/91/prepub
